# Activity of Indatuximab Ravtansine against Triple-Negative Breast Cancer in Preclinical Tumor Models

**DOI:** 10.1007/s11095-018-2400-y

**Published:** 2018-04-17

**Authors:** Kurt Schönfeld, Peter Herbener, Chantal Zuber, Thomas Häder, Katrin Bernöster, Christoph Uherek, Jörg Schüttrumpf

**Affiliations:** 1 Corporate Research & Development, Biotest AG, Landsteinerstraße 5, 63303 Dreieich, Germany; 2 Corporate Project & Portfolio Management, Biotest AG, Landsteinerstraße 5, 63303 Dreieich, Germany

**Keywords:** CD138, Indatuximab ravtansine, Remission, Triple-negative breast cancer, Tumor regression

## Abstract

**Purpose:**

Triple-negative breast cancer (TNBC) is related with a poor prognosis as patients do hardly benefit from approved therapies. CD138 (Syndecan-1) is upregulated on human breast cancers. Indatuximab ravtansine (BT062) is an antibody-drug-conjugate that specifically targets CD138-expressing cells and has previously shown clinical activity in multiple myeloma. Here we show indatuximab ravtansine as a potential mono- and combination therapy for TNBC.

**Methods:**

The effects of indatuximab ravtansine were assessed *in vitro* in SK-BR-3 and T47D breast cancer cell lines. The *in vivo* effects of indatuximab ravtansine alone and in combination with docetaxel or paclitaxel were assessed in MAXF401, MAXF1384 and MAXF1322 xenograft TNBC models.

**Results:**

CD138^+^ SK-BR-3 and T47D cells were highly sensitive to indatuximab ravtansine. The high CD138-expressing MAXF401 xenograft model demonstrated strong inhibition of tumor growth with 4 mg/kg indatuximab ravtansine. High doses of indatuximab ravtansine (8 mg/kg), docetaxel and the combination of both led to complete remission. In the low CD138-expressing MAXF1384 xenograft model, only combination of indatuximab ravtansine and docetaxel demonstrated a significant efficacy. In the MAXF1322 xenograft model, indatuximab ravtansine alone and in combination with paclitaxel elicited complete remission.

**Conclusions:**

These data demonstrate potential use of indatuximab ravtansine in combination with docetaxel or paclitaxel for CD138-positive TNBC.

## Introduction

Breast cancer represents the most common non-dermatological malignant neoplasm in women and, after lung cancer, it is the second leading cause of cancer-related death among women. In 2016, approximately 40,000 women in the United States died from breast cancer [1], and 92,300 were predicted to die from the disease in Europe [2]. Triple-negative breast cancer (TNBC), in which the estrogen receptor (ER), progesterone receptor (PR), and human epidermal growth factor receptor 2 (HER2) are not expressed [3, 4], accounts for approximately 170,000 of the 1 million breast cancer cases which are diagnosed worldwide annually [4].

The lack of target receptors (ER, PR, and HER2) means that patients with TNBC do not benefit from hormonal or anti-HER2-based therapy [4]. Moreover, although chemotherapy represents the mainstay of systemic treatment, patients with advanced disease typically respond poorly and rapidly progress [3]. The lack of targeted therapies and the poor prognosis of TNBC means that clinical research in the area of TNBC is of great importance [4].

The ability of tumor cells to invade and metastasize relies on sequential adhesion, motility, and proliferation [5]. Heparan sulfate proteoglycans (HSPGs) are a class of molecules that can influence each of these essential steps [5]. CD138 (syndecan-1) is the most characterized member of the syndecan family, which constitute a major class of cell surface HSPGs [5]. CD138 acts as a receptor for the extracellular matrix and is involved in several cellular functions including cell-cell adhesion, cell-matrix interaction, cellular proliferation, and differentiation [6, 7]. CD138 is overexpressed on malignant plasma cells and expressed by epithelial cells in non-hematopoietic tissue, although transient expression in condensing mesenchyme also occurs during embryonal morphogenesis [7, 8]. Expression of CD138 is widely detected in epithelial neoplasms of various origins and, in rare cases, mesenchymal tumors. Certain pathophysiologic conditions such as tumorigenesis, neoplastic progression, and metastasis can alter the expression of CD138 and its family members [7]. Studies in human breast cancer samples have shown CD138 to be upregulated compared to normal breast tissues, including TNBC which exhibit markedly higher CD138 levels compared to luminal subtypes [7]. Furthermore, CD138 has been reported as a marker of poor prognosis in patients with breast cancer [5].

Indatuximab ravtansine (BT062) is an antibody-drug conjugate based on a murine/human chimeric form of B-B4 (CD138-specific antibody), which is covalently conjugated to the maytansinoid drug DM4 via a disulfide bond-based linker [6]. Maytansinoids are structural analogs of the cytotoxic agent maytansine, which has been evaluated in phase I and phase II clinical trials [9–13]. They exhibit anti-mitotic activity by inducing metaphase arrest of dividing cells, causing cell death [14]. Upon internalization of indatuximab ravtansine by target tumor cells, lysosomal processing of the disulfide linker generates a lysine metabolite which is reduced and S-methylated producing the lipophilic and cytotoxic metabolite, S-methyl-DM4 [6, 15]. The mechanism of action of indatuximab ravtansine resembles that of trastuzumab emtansine, an antibody-drug conjugate indicated for HER2-positive metastatic breast cancer which uses the HER2-targeting properties of trastuzumab to deliver the cytotoxic DM1 within the cell by means of a stable linker [16]. The intracellular drug delivery to target tumor cells can improve the therapeutic index of the cytotoxic drug and minimize exposure of normal tissue [16].

Indatuximab ravtansine has previously been shown *in vivo* in multiple myeloma mouse xenograft models to significantly inhibit tumor growth and prolong host survival without any toxicity signals [6]. Based on these preclinical results, a phase I clinical trial of indatuximab ravtansine demonstrated the first signs of clinical activity in patients with relapsed or refractory multiple myeloma without any toxicity signals [17]. Furthermore, preliminary data from a phase I/IIa study in relapsed or refractory multiple myeloma indicated that indatuximab ravtansine is well tolerated even in a multiple-dose schedule and provided further evidence of clinical activity [18].

To further examine the potential role of indatuximab ravtansine in TNBC, the *in vitro* mechanistic actions in breast cancer cell lines and its anti-tumor effects in xenograft TNBC models were investigated as monotherapy, and in combination with either docetaxel or paclitaxel.

## Methods

### CD138 Expression in Primary and Metastatic Breast Cancer Tissues

Immunohistochemical (IHC) staining was performed on TNBC (ER–/PR–/HER2–negative) samples to determine levels of CD138 expression. Paraffin blocks of TNBC samples were purchased from three sources (Pantomics #BRM481, Tristar #69571112, Tristar #69572306) and cut into 5 μm sections. A total of 19 primary breast cancer samples and 42 metastatic breast cancer samples were tested. Samples were mounted on positively-charged glass slides with a maximum of three samples from three different tissues per slide. All experiments included normal human colon and tonsil sections (normal tissue controls) (Ardais Corp, Cooperative Human Tissue Network, Cytomyx, Mercy Health Systems) and HSC4 (JCRB Cell Bank, Japan), ACHN, and T47D (American Type Culture Collection) cells (positive controls) as well as the test samples.

### CD138 Expression in Patient-Derived Xenograft Samples

To confirm CD138 expression in breast cancer xenograft samples, eight samples each from the MAXF1384 (Oncotest), MAXF401 (Oncotest), and MAXF1322 (Oncotest) TNBC xenograft models treated with indatuximab ravtansine, phosphate buffered saline (PBS) or docetaxel were paraffin embedded and assessed by IHC and hematoxylin and eosin (H&E).

Automated IHC staining was performed with the monoclonal mouse anti-CD138 antibody MI15 (Dako) or an isotype control antibody (immunoglobulin IgG1), each diluted in Leica antibody diluent (DakoCytomation) to a concentration of 0.25 μg/mL. In addition, all tissue samples were evaluated by H&E staining to determine anatomic site, tumor type and tissue integrity.

#### Immunohistochemistry Assay

**Fig. 1:**
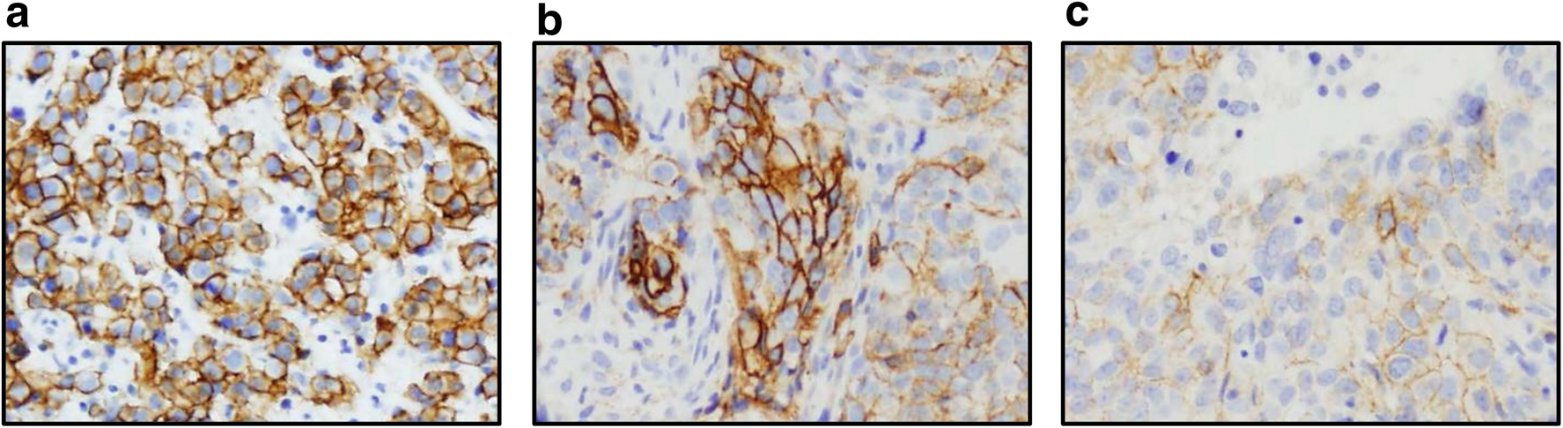
**Representative CD138 staining in TNBC samples (20X magnification)**. (**a**) Level 3 homogeneous staining, (**b**) level 2–3 heterogeneous staining, and (**c**) level 1–2 homogeneous staining.

Staining intensity and proportion of cells stained in each sample type were determined by a Board-certified pathologist. Membrane-associated staining was recorded for all samples and any staining patterns noted. Final scores relate to membrane staining; if only cytoplasmic staining was determined, the final score was reported as zero. Samples were categorized according to their intensity and uniformity as shown in Table I. Examples of the three staining levels are shown in Fig. 1. If there were duplicate results from one patient (i.e. one tissue sample), only the higher score was included in analysis.

**Table I:** IHC scoring system and categories

**Uniformity (proportion of stained cells)**		**Intensity (brightness of stain)**		**Intensity category**
0	Negative	0	Negative	Negative
Focal	<25%	0–1	Very weak	Weak
Heterogeneous (hetero)	25–75%	1	Weak	
Homogeneous (homo)	>75%	1–2	Weak to moderate	
		2	Moderate	Moderate
		2–3	Moderate to strong	Strong
		3	Strong

### CD138 Expression and Sensitivity to Indatuximab Ravtansine in Breast Cancer Cell Lines

In order to evaluate the binding and activity of indatuximab ravtansine against breast cancer cell lines, the CD138-positive cell lines SK-BR-3 (American Type Culture Collection) and T47D (American Type Culture Collection) were detached from cell culture flasks by Accutase treatment before incubation of 1 × 10^5^ cells with nBT062, the anti-CD138 antibody moiety of the conjugate indatuximab ravtansine.

nBT062 was diluted in 100 μL at concentrations of 0.98–125 ng/mL. Cells were washed twice and bound nBT062 antibody was detected by fluorescein isothiocyanate (FITC) fluorescence after incubation of the cells with 1/50 diluted FITC-conjugated goat anti-human IgG (Immunotech) in PBS using a fluorescence-activated cell sorting (FACS) Calibur flow cytometer (Beckton Dickinson). All determinations were performed in duplicate samples. Relative fluorescence was calculated using the mean intensity of tested samples with the mean fluorescence of negative controls; untreated cells, isotype control treated cells, and cells treated with the secondary antibody only.

In order to determine sensitivity to indatuximab ravtansine, breast cancer cells at a density of 900 cells/well, were seeded into 96-well plates and incubated at 37°C for 24 h. The active immunoconjugate indatuximab ravtansine was added to wells in triplicate at concentrations of 0.01–100 nM, depending on the cell type, and incubated. After 5 days, 10 μL of WST-1 reagent containing tetrazolium salt (Roche) was added and incubated for 3 h and cell viability was quantified by measuring absorbance at 450 nm *versus* 690 nm (reference wavelength) in a microplate reader.

### Internalization of Indatuximab Ravtansine into Breast Cancer Cell Lines

SK-BR-3 cells were seeded on NEXITRON coverslips (Schott Technical Glass Solutions GmbH) and cultivated for 48 h in complete culture medium. Cells were subsequently incubated for 30 min at 30°C with 20 μg/mL of fluorescently labelled (Dylight 488) nBT062 or the immunoconjugate indatuximab ravtansine. Cells were washed three times with Hank’s balanced salt solution (HBSS) and cultivated in complete culture medium for 0–72 h.

Localization of nBT062 and indatuximab ravtansine was assessed by IHC in cells fixed by incubation in 4% paraformaldehyde (PFA)/PBS at room temperature for 15 min, washed in PBS, permeabilized in 0.02% Saponin/PBS for 1 min and subsequently blocked by incubation in 1× BMB/PBS for 10 min (Boehringer Blocking Reagent, Roche). Distribution of fluorescence was assessed by treatment of the cells with fluorescent labelled goat anti-human antibody (Dianova) and incubated at room temperature for 2 h. Cells were washed in PBS and stained in 200 μl CellMask™ Orange Plasma Membrane Stain (Life Technologies)/DRAQ5 solution (1 μg/mL) at room temperature for 15 min. Cells were washed in PBS and embedded on microscope slides with DAKO fluorescent mounting medium.

The time course of internalization into SK-BR-3 cells was determined by microscopic analysis. Cells were stained in 200 μL CellMask™ Orange Plasma Membrane Stain (Life Technologies)/DRAQ5 solution for 15 min at 37°C, washed in HBSS and fixed by incubation in 4% PFA/PBS at room temperature for 15 min and embedded with DAKO fluorescent mounting medium.

Analysis of the subcellular localization of nBT062 and indatuximab ravtansine (both Dylight 488 labelled) was undertaken by fixing SK-BR-3 cells at 40 min, 1, 2, and 4 h after initial antibody binding and subjected to IHC staining against early endosomal antigen-1 (EEA-1) (rabbit anti-EEA1 antibody, Abcam) or lysosomal-associated membrane protein (LAMP) (rabbit anti-LAMP antibody, Abcam). They were added to permeabilized cells and incubated at room temperature for 1 h. Cells were washed and secondary antibody (goat anti-rabbit Cy3, Dianova), diluted 1:500 was added for 2 h at room temperature. Cells were washed in PBS and stained for cell nuclei in DRAQ5 solution for 15 min at room temperature, followed by wash in PBS and embedding on microscope slides with DAKO fluorescent mounting medium.

### ***In Vivo*** Effect of Indatuximab Ravtansine in Xenograft Models

All animal experiments were performed at Oncotest GmbH (Freiburg, Germany). Experiments were approved by the Committee on the Ethics of Animal Experiments of the Regierungspräsidium Freiburg (Freiburg, Germany; permit number: G-13/13) and conducted according to the guidelines of the German Animal Welfare Act (Tierschutzgesetz) and “Principles of Laboratory Animal Care” (NIH publication #85–23, revised in 1985).

NMRI nude mice were inoculated subcutaneously (SC) with tumor fragments from the low CD138-expressing MAXF1384 or the high CD138-expressing MAXF401 xenografts (Oncotest). Xenografts were passaged three to five times, until there were clear signs of tumor growth.

Animals bearing at least one tumor of 50–250 mm^3^ (preferably 80–200 mm^3^) were randomized to one study arm (Table II). All drugs were given intravenously (IV) once-weekly over a 5 week cycle. Mortality was assessed daily, and body weight (calculated as relative to day 0) and absolute tumor volume twice-weekly. Tumor volume was calculated as (A x B^2^) × 0.5, where A was the largest and B the perpendicular tumor diameter; tumor volume was calculated relative to day 0.

**Table II:** Study design/dosing in xenograft models MAXF1384 and MAXF401

**Compound**	**Dose**	**Dosing days**	**Number of test animals (per xenograft model**)
Saline	10 mL/kg	0,7,14,21,28,35	8
Docetaxel	10 mg/kg	0,7,14	8
Indatuximab ravtansine	1 mg/kg	0,7,14,21,28,35	8
Indatuximab ravtansine	2 mg/kg	0,7,14,21,28,35	8
Indatuximab ravtansine	4 mg/kg	0,7,14,21,28,35	8
Indatuximab ravtansine	8 mg/kg	0,7,14,21,28,35	8
Indatuximab ravtansine + docetaxel	2 mg/kg 10 mg/kg	0,7,14,21,28,35 0,7,14	8

Relative tumor volume was used to calculate tumor doubling time and tumor inhibition (median relative tumor volume compared with the control group) classified according to predefined criteria (Table III).

**Table III:** Tumor control efficacy criteria

**Classification**	T/C*
Inactive	≥65%
Borderline	50% – <65%
Moderate	25% – <50%
High	10% – <25%
Very high	5% – <10%
Complete remission	<5%

*T/C = median tumor volume in test group/median tumor volume in control group

#### Statistical Analysis

Statistical analysis of tumor inhibition was by the non-parametric Kruskal-Wallis test followed by Dunn’s multiple comparison test. Significance for time to tumor inhibition was tested by log-rank Mantel-Cox for pairwise comparisons. Correlation between tumor control and indatuximab ravtansine was analyzed by Spearman correlation analysis with predefined r-values to indicate no, moderate, high, or very high correlation. Significance was determined by conventional *p*-value of *p* ≤ 0.05.

In addition, a second study was undertaken in a third xenograft model, MAXF1322, to evaluate the effects of indatuximab ravtansine as monotherapy and in combination with paclitaxel (Table IV). Efficacy was assessed according to tumor volume and maximal tumor inhibition as described in Table III.

**Table IV:** Study design/dosing in xenograft model MAXF1322

Compound	Dose	Dosing days	Number of test animals
Saline	10 mL/kg	0,7,14,21,28,35	9
Paclitaxel	10 mg/kg	1,8,15,22	7
Indatuximab ravtansine	0.5 mg/kg	0,7,14,21,28,35	7
Indatuximab ravtansine	1 mg/kg	0,7,14,21,28,35	7
Indatuximab ravtansine	2 mg/kg	0,7,14,21,28,35	7
Indatuximab ravtansine	4 mg/kg	0,7,14,21,28,35	7
Indatuximab ravtansine + paclitaxel	1 mg/kg 10 mg/kg	0,7,14,21 1,8,15,22	5
Indatuximab ravtansine + paclitaxel	4 mg/kg 10 mg/kg	0,7,14,21,28,35 1,8,15,22	7
nBT062	4 mg/kg	0,7,14,21,28,35	9
nBT062 + paclitaxel	4/2/4 mg/kg 10 mg/kg	0,7/14,21/28,35 1,8,15,22	7
nBT062 + DM4	4 mg/kg 75 μg/kg	0,7,14,21 0,7,14,21	4
DM4	75 μg/kg	0,7,14,21	4

## Results

### CD138 Expression in TNBC Samples

A high proportion of primary breast cancer samples expressed CD138 (16/19; 84%) by IHC, however, the majority of these had a weak staining score (Table V). Similarly, the majority of metastatic breast cancer samples also expressed CD138 (33/42; 79%), however, a higher proportion had moderate or strong IHC staining (Table V). Representative photos of CD138 IHC in TNBC are provided (Fig. 1).

**Table V:** CD138 expression evaluated by IHC in 61 TNBC tumor microarrays

Intensity category	Primary tumor samples (*n* = 19), Total (%)	Metastatic tumor samples (*n* = 42), Total (%)
Negative	3 (16%)	9 (22%)
Weak	14 (74%)	14 (33%)
Moderate	2 (10%)	3 (7%)
Strong	0 (0%)	16 (38%)

### Binding of, and Sensitivity to, Indatuximab Ravtansine against Breast Cancer Cells Lines

The binding of nBT062 and resulting characterization of CD138 expression indicated variable expression of CD138 between the two cell lines tested; notably, expression was 2-fold higher in the SK-BR-3 cell line compared with the T47D line (Table VI). Moreover, SK-BR-3 cells were more sensitive to indatuximab ravtansine as demonstrated by the median inhibitory concentration (IC_50_) values (Table VI).

**Table VI:** CD138 expression and sensitivity in SK-BR-3 and T47D cell lines

	CD138 expression: Mean relative fluorescence intensity (number of tests)	Sensitivity to indatuximab ravtansine:Mean IC_50_ [nM] (number of tests)
SK-BR-3	485 (4)	2.72 (7)
T47D	217 (4)	89.28 (4)

Sensitivity of the two breast cancer cell lines to indatuximab ravtansine was also studied. Mean IC_50_ values indicate that the SK-BR-3 cell line (IC_50_ 2.72 nM) was more sensitive to indatuximab ravtansine than T47D cells (IC_50_ 89.28 nM), but sensitivity did not directly correlate with CD138 expression level (i.e. T47D cells were approximately 33-fold less sensitive to indatuximab ravtansine than SK-BR-3 cells but CD138 expression levels were only around 2-fold lower in the T47D compared to SK-BR-3 cells). On the basis of these results, the indatuximab ravtansine-sensitive breast cancer cell line SK-BR-3 was chosen to analyze indatuximab ravtansine internalization properties into cells.

### Internalization of nBT062 and Indatuximab Ravtansine

**Fig. 2:**
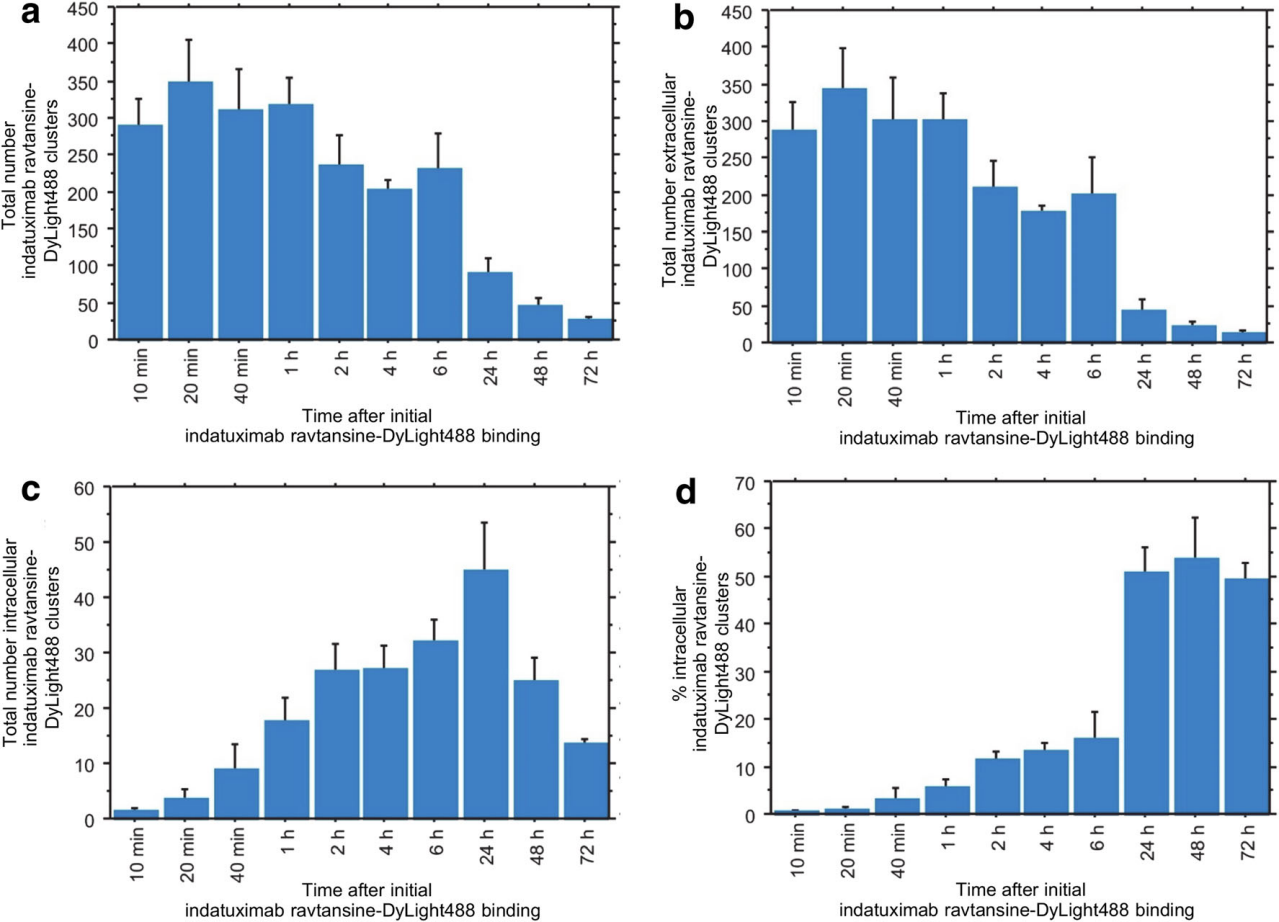
**Time dependent distribution of fluorescently labelled indatuximab ravtansine in SK-BR-3 cells**. Quantification of (**a**) total number of indatuximab ravtansine-DyLight488 clusters, (**b**) total number of extracellular indatuximab ravtansine-DyLight488 clusters, (**c**) total number of intracellular indatuximab ravtansine-DyLight488 clusters, and (**d**) percentage of intracellular indatuximab ravtansine-DyLight488 clusters in relation to total cluster number. Error bars, SEM; *n* = 3 for all groups.

Time-dependent analysis of the distribution of indatuximab ravtansine confirmed internalization, with a decrease in extracellular and increase in intracellular localization, so that at 24 h approximately 50% of the antibody-conjugate was internalized (Fig. 2).

**Fig. 3:**
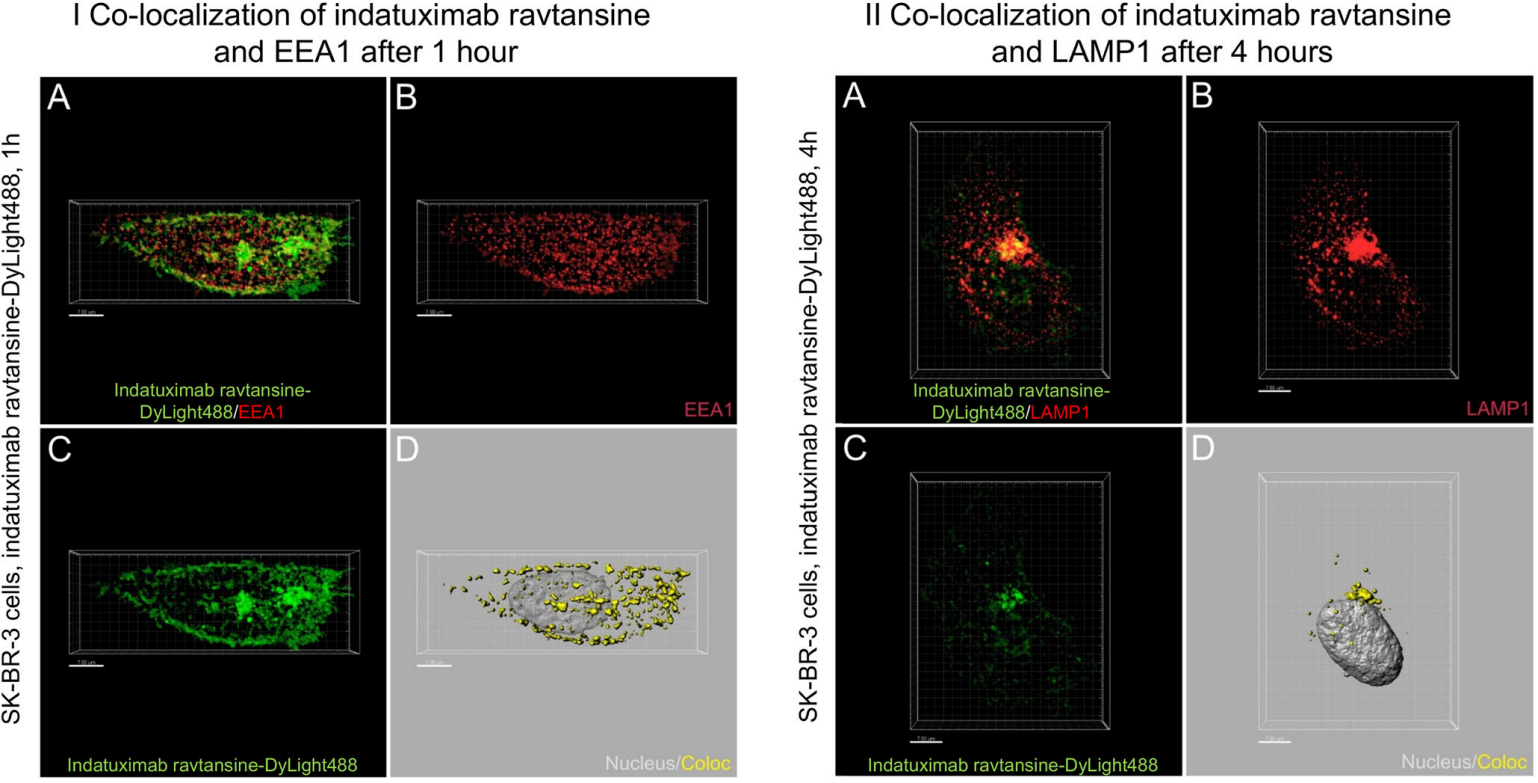
**Internalization of indatuximab ravtansine through the endosomal (I) and lysosomal (II) pathway in SK-BR-3 cells**. Grouped panels represent analyses at 1 and 4 h after initial antibody binding. (**a**) Merged DyLight488 (green, indatuximab ravtansine) and Cy3 (red, EEA1 [I]; LAMP1 [II]) fluorescent z-stack recordings. (**b**) Cy3 (EEA1 [I]; LAMP1 [II]) fluorescent z-stack recordings. (**c**) DyLight488 (indatuximab ravtansine) fluorescent z-stack recordings. (**d**) 3-dimensional reconstruction of cell nucleus (light grey, nucleus) and portions of DyLight488 fluorescence co-localized to EEA1-postive endosomes [I]; LAMP1-postive lysosomes [II] (yellow, Coloc).

Distinct cytoplasmic localization of fluorescently labelled nBT062 and indatuximab ravtansine clusters were observed based on EEA1 and LAMP1 staining, with evidence for this internalization via the endolysosomal pathway (Fig. 3).

### CD138 Expression in TNBC Xenograft Models

**Fig. 4:**
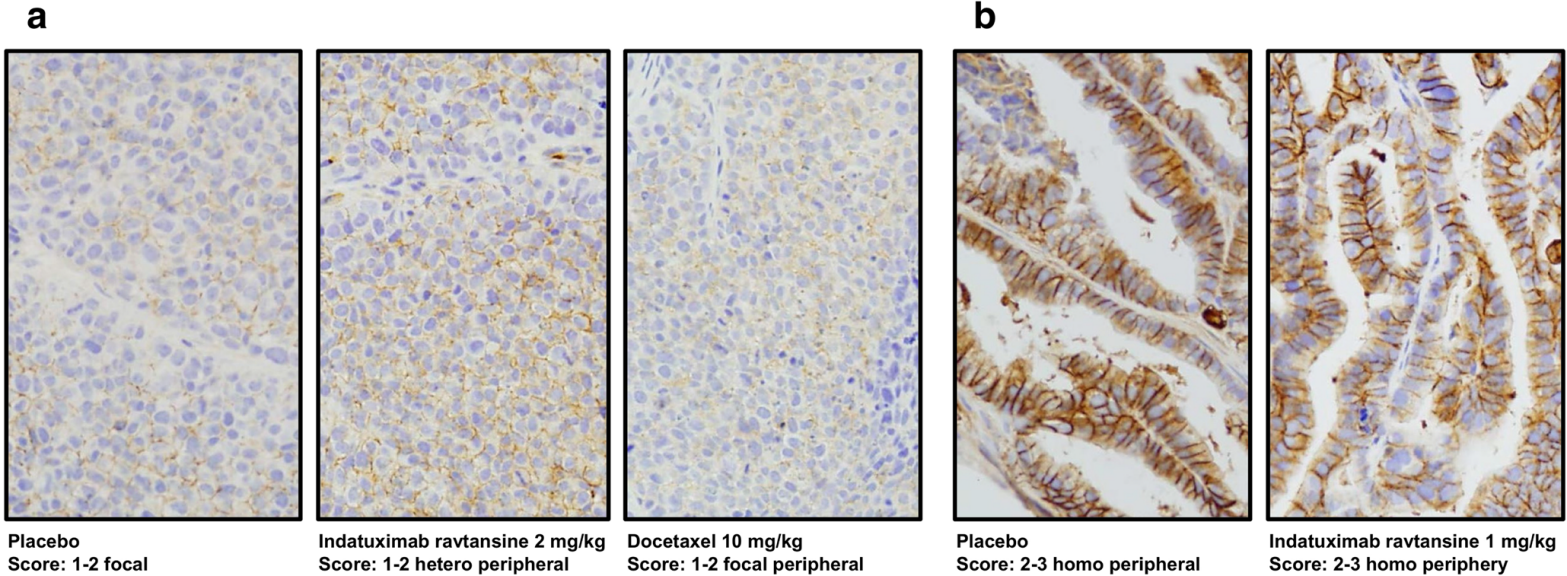
**Representative staining of CD138**. IHC CD138 staining in (**a**) MAXF1384 xenograft model with low CD138 expression treated with placebo (PBS), 2 mg/kg indatuximab ravtansine or 10 mg/kg docetaxel and (**b**) MAXF401 xenograft model with high CD138 expression treated with placebo (PBS) or 1 mg/kg indatuximab ravtansine, using monoclonal mouse anti-CD138 antibody and H&E.

IHC analysis of indatuximab ravtansine-treated xenograft mouse models confirmed one with weak CD138 expression (MAXF1384) and one with strong expression (MAXF401) (Fig. 4).

### ***In Vivo*** Effect of Indatuximab Ravtansine in Xenograft Models

**Fig. 5:**
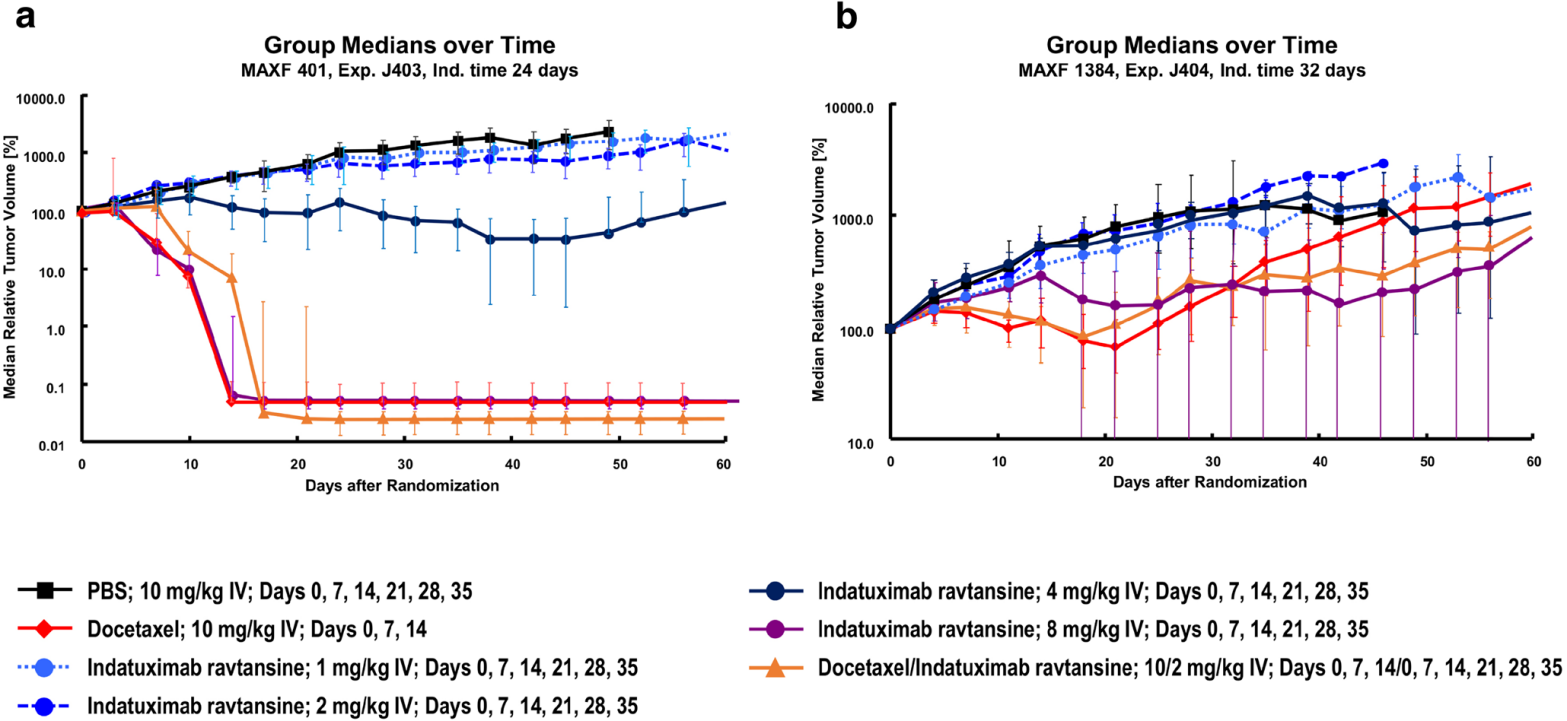
**Tumor growth curves (relative tumor volume) in MAXF401 and MAXF1384 xenograft models**. Median relative tumor volume (%) was assessed in (**a**) high CD138-expressing MAXF401 xenograft model and (**b**) low CD138-expressing MAXF1384 xenograft model with different doses of indatuximab ravtansine and docetaxel.

In both the low CD138-expressing xenograft (MAXF1384) and high-expressing xenograft (MAXF401) mouse models, biologic activity of indatuximab ravtansine was demonstrated (Fig. 5); although this effect was most notable in the xenograft (MAXF401) with high levels of CD138 expression (Fig. 5a). In this model, relative tumor volumes indicated strong inhibition of growth with indatuximab ravtansine 4 mg/kg while 8 mg/kg and the combination of indatuximab ravtansine and docetaxel were significantly superior to control. Docetaxel also demonstrated a significant effect on tumor growth (Fig. 5a). Monotherapy with indatuximab ravtansine 8 mg/kg, docetaxel, and the combination of indatuximab ravtansine and docetaxel led to complete remissions, and according to pre-defined criteria for response (Table III), indatuximab ravtansine 4 mg/kg was also highly effective (Table IV).

For the xenograft with low expression of CD138, only the combination of indatuximab ravtansine and docetaxel was significantly superior to control (Fig. 5b), and this regimen had high and very high anti-tumor activity with minimum T/C values of 16.3% and 8.3%, respectively. However, these data also indicated that at higher doses (8 mg/kg), indatuximab ravtansine monotherapy had clinical activity even in tumors with a weak CD138 expression.

**Fig. 6:**
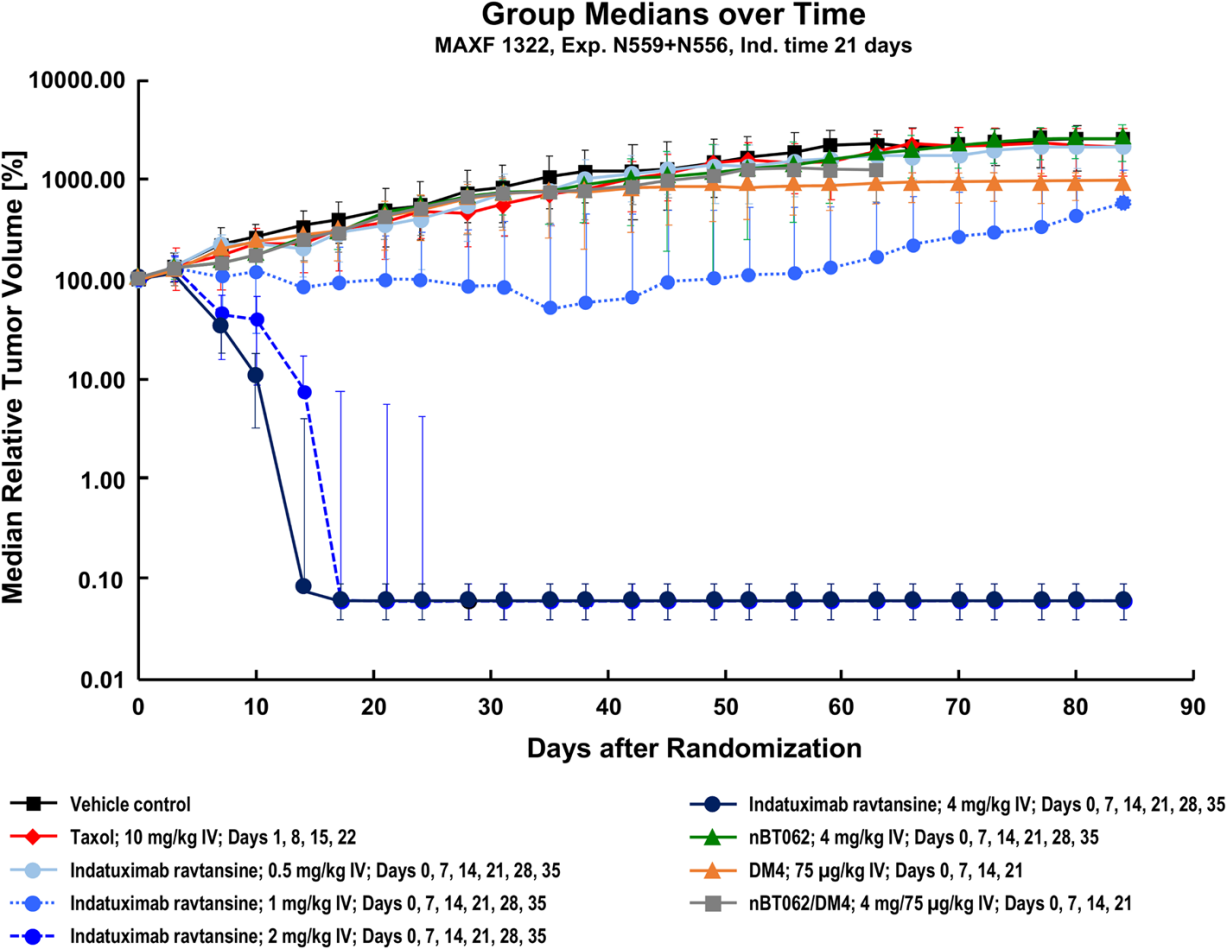
**Tumor growth curves (relative tumor volume) in MAXF1322 xenograft model**. Median relative tumor volume (%) was assessed in the MAXF1322 xenograft model with different doses of indatuximab ravtansine, paclitaxel (taxol), DM4 and nBT062.

In addition, using the MAXF1322 model, it was demonstrated that the anti-CD138 antibody nBT062 had no effect on tumor growth. Notably, this model was also resistant to paclitaxel and relatively resistant to DM4 monotherapy. The addition of anti-CD138 antibody to DM4 had no effect on efficacy (Fig. 6), however, the conjugated antibody indatuximab ravtansine 2 mg/kg and 4 mg/kg demonstrated anti-tumor activity as monotherapy. The combination of indatuximab ravtansine and paclitaxel was also active. This was confirmed by the tumor control data, where the combination of paclitaxel with indatuximab ravtansine at the highest dose (4 mg/kg) resulted in complete remission, with an optimal T/C value of 0%. The combination of paclitaxel with only 1 mg/kg indatuximab ravtansine resulted in an optimal T/C value of 11.3%.

## Discussion

Over the last few years, great strides have been made in breast cancer therapy, however much of this progress has been confined to those patients who overexpress HER2 [3]. TNBC is an aggressive subtype of breast cancer with generally poor prognosis and few available treatment options, which therefore represents a challenge for both patients and clinicians [4, 19].

CD138 is a cell surface integral membrane protein [6], which is variably expressed in a variety of cancers and may play a significant role in mammary carcinogenesis [7]. In a cohort of breast carcinomas with associated distant metastasis, CD138 expression was associated with a higher histologic grade and inversely related to hormonal receptor status [7]. Compared to luminal subtypes, the HER2-positive subtype and TNBC exhibited markedly higher CD138 levels. High expression of CD138 was associated with a negative impact on both overall and disease-free survival [7]. In this study, a high proportion of primary and metastatic TNBC samples expressed CD138 (84% and 79%, respectively) and a higher proportion of metastatic samples had moderate or strong staining in the non-validated IHC assay conducted under GLP standards. The metastatic setting may therefore be the preferred option for subsequent clinical studies of CD138-targeted therapy in TNBC.

Indatuximab ravtansine is an antibody-drug conjugate consisting of an anti-CD138 antibody and the cytotoxic maytansinoid agent DM4. Indatuximab ravtansine exerts its anti-tumor effects in a similar manner to trastuzumab emtansine [16]. It specifically binds to CD138-positive cancer cells. Once internalized, a lysine metabolite is generated and DM4 is released, destroying the cancer cell [6, 15]. Studies using multiple myeloma cells have provided a useful insight into the mechanistic activity of indatuximab ravtansine as well as its anti-tumor properties. *In vitro* studies have shown indatuximab ravtansine to inhibit the proliferation of multiple myeloma cells through induction of G_2_-M cell cycle arrest followed by apoptotic cell death. This was evidenced by dose-dependent cleavage of caspase-8, caspase-9, caspase-3, and poly (ADP-ribose) polymerase (PARP), as well as increased Apo 2.7-positive cells [6]. In this study with breast cancer cell lines, distinct cytoplasmic localization of fluorescently labelled nBT062 and indatuximab ravtansine clusters were also observed.

Previous studies with indatuximab ravtansine in multiple myeloma mouse xenograft models showed significant tumor growth inhibition and prolonged host survival but minimal anti-tumor activity with the unconjugated antibody [6]. Likewise, in this study the unconjugated antibody had no effects on tumor growth in the *in vivo* MAXF1322 xenograft model. Similarly, the addition of DM4 had no effect on efficacy, however the conjugated antibody indatuximab ravtansine at 2 mg/kg and 4 mg/kg demonstrated anti-tumor activity both as monotherapy and in combination with paclitaxel. The anti-tumor activity of indatuximab ravtansine was also observed in the low CD138-expressing xenograft MAXF1384; and the high-expressing xenograft MAXF401 when used in combination with docetaxel. Only in xenograft model MAXF1384 was indatuximab ravtansine less effective as monotherapy, indicating that higher CD138 expression may be associated with better efficacy.

Rousseau *et al*. have also shown favorable results of CD138 targeted radioimmunotherapy: Human MDA-MB-468 TNBC bearing mice were treated with the murine anti-CD138 antibody B-B4 radiolabeled with ^131^I. In line with the results from our study, they observed a significant reduction of tumor growth in ^131^I-B-B4 treated animals compared to control, despite CD138-expression was low on MDA-MB-468 cells, and thus confirming CD138 as suitable target for TNBC therapy [20].

In conclusion, these preclinical data show promising results for the use of the antibody drug-conjugate indatuximab ravtansine for the treatment of patients with TNBC-expressing CD138. In patients with lower expression of CD138, combination with taxanes, such as paclitaxel and docetaxel, may be considered.

## Abbreviations

EEA-1: Endosomal antigen-1
ER: Estrogen receptor
FACS: Fluorescence-activated cell sorting
FITC: Fluorescein isothiocyanate
H&E: Hematoxylin and eosin
HBSS: Hank’s balanced salt solution
HER2: Human epidermal growth factor receptor 2
HSPGs: Heparan sulfate proteoglycans
IC_50_: Inhibitory concentration
IHC: Immunohistochemical
IV: Intravenously
LAMP: Lysosomal-associated membrane protein
PARP: Poly ADP-ribose polymerase
PBS: Phosphate buffered saline
PFA: Paraformaldehyde
PR: Progesterone receptor
SC: Subcutaneously
SEM: Standard error of the mean
TNBC: Triple-negative breast cancer
